# Comparative anatomical studies of some *Teucrium* sect. *Teucrium* species: *Teucrium alyssifolium* Stapf, *Teucrium brevifolium* Schreb. and *Teucrium pestalozzae* Boiss. (Lamiaceae)

**DOI:** 10.3897/phytokeys.96.24498

**Published:** 2018-04-03

**Authors:** Gülay Ecevit-Genç, Betül Büyükkılıç-Altınbaşak, Taner Özcan, Tuncay Dirmenci

**Affiliations:** 1 Istanbul University, Faculty of Pharmacy, Department of Pharmaceutical Botany Istanbul, Turkey; 2 Bezmialem Vakıf University, Faculty of Pharmacy, Department of Pharmaceutical Botany Istanbul, Turkey; 3 Balıkesir University, Faculty of Necatibey Education, Department of Biology Education Balıkesir, Turkey

**Keywords:** Lamiaceae, *Teucrium*, leaf anatomy, stem anatomy

## Abstract

*Teucrium alyssifolium* Stapf (endemic), *Teucrium pestalozzae* Boiss. (endemic) and *Teucrium brevifolium* Schreb. are three closely related taxa in Teucrium sect. Teucrium. The obtained data from the anatomical studies revealed that these three taxa represent the general anatomical characteristics of the Lamiaceae family. Leaves, anatomical features such as thick cuticle, abundant trichomes, rich palisade parenchyma layer in the mesophyll provide evidence that these three species are xeromorphic structures. Leaf and stem anatomy showed that the taxa have generally similar anatomical features. However, cuticle layers, epidermis cells size, indumentum density, mesophyll types, palisade parenchyma occupied in the mesophyll, presence of spherocrystals in leaves and parenchyma, collenchyma and sclerenchyma layers in stems show differences amongst the taxa. Anatomical characters of leaf and stem of these taxa are examined for the first time in this study.

## Introduction

The genus *Teucrium* L. has approximately 300 species all over the world. *Teucrium*’s cosmopolitan distribution is mainly concentrated in Europe, North Africa and in the temperate parts of Asia (Ecevit-Genç et al. 2015, 2017). *Teucrium* is a large and polymorphic genus which is represented by 49 taxa (36 species) in Turkey. There are 18 endemic taxa (Govaerts 1999, Duman 2000, Dönmez 2006, Parolly and Eren 2007, Dönmez et al. 2010, Dinç et al. 2011a, 2011b, Dirmenci 2012, Özcan et al. 2015a, Vural et al. 2015, Dinç and Doğu 2016). The classification of *Teucrium* is based on sections. The main characters in the separation of sections are the calyx-shape and flower arrangement (Davis 1982; Navarro et al. 2004; Dinç et al. 2008; Özcan et al. 2015a; Vural et al. 2015). Especially, leaf anatomy is important for the classification of the genus (Dinç et al. 2009). Also, absence or presence of trichomes and their types on the nutlets and vegetative parts are very important for classifying the species (Dinç et al. 2011a, Ecevit-Genç et al. 2015, 2017).

*Teucrium* species have traditionally been used in Turkey for abdominal pain, stomach-ache, common cold, high fever, antipyretic, rheumatic pain and as an antidiabetic (Sezik et al. 2001, Aksoy-Sagirli et al. 2015).

*T. alyssifolium* is a narrowly distributed endemic species. It is classified as a ‘Conservation Dependent (LR/cd)’ category of IUCN and it is a source of polyphenols and flavonoids and has confirmed antioxidant activities (Semiz et al. 2016). *T. pestalozzae* is an endemic species and its essential oil is characterised with β-caryophyllene (27.6%) and germacrene D (13.8%) as major constituents (Baser et al. 1997). Spathulenol and δ-cadinene are the main compounds of *T. brevifolium* essential oil and it has shown anti-tumour activities, a selective cytotoxicity on large lung carcinoma (IC_50_ value of 80.7 μg/ml) (Menichini et al. 2009).

The chromosome numbers are reported as 2n = 10, 14, 16, 18, 22, 26, 28, 30, 32, 36, 39, 48, 52, 56, 58, 60, 62, 64, 78, 80, 86, 90, 96 and 104 in the genus *Teucrium* (http://www.tropicos.org/Project/IPCN). The chromosome number of *T. brevifolium* examined in this study was determined as 2n = 30. Another member of the sect. Teucrium, *T. sandrasicum*, was studied and it was determined that the chromosome number is the same as *T. brevifolium* (Özcan et al. 2015b).

Pollen morphology supplies useful data at the taxonomic level in *Teucrium* (Navarro et al. 2004, Marzouk et al. 2017). Oybak and İnceoğlu (1988) studied pollen morphology of some Turkish *Teucrium* members. They found out that the species belonging to different sections had different pollen type and pollen shape, while pollen grain size and apocolpia size were the main characters used for distinguishing the species. Especially, *T. alyssifolium* could be easily separated from the other species of the sect. Teucrium according to pollen data.

There are several studies on *Teucrium* anatomy (Lakusic et al. 2006, 2010, Dinç et al. 2008, 2009, 2011a, 2011b, Dehshiri and Azadbakht 2012, Dinç and Doğu 2012, Doğu et al. 2013, Özcan 2013, Özcan and Eminagaoglu 2014, Ruiters et al. 2016). However, the anatomy of *T. pestalozzae*, *T. brevifolium* and *T. alyssifolium* has not been investigated. In our previous studies, we investigated the nutlet and leaf micromorphology of some species belonging to the sect. Teucrium in Turkey (Ecevit-Genç et al. 2015, 2017). In the present study, we report on the anatomical features of the leaves and stems of *T. alyssifolium*, *T. brevifolium* and *T. pestalozzae*. The aim of this paper is to understand the anatomy of these three *Teucrium* species. Also, a better understanding of systematics helps the distinction of morphologically closely related taxa from each other.

## Materials and methods

**Figure 1.**
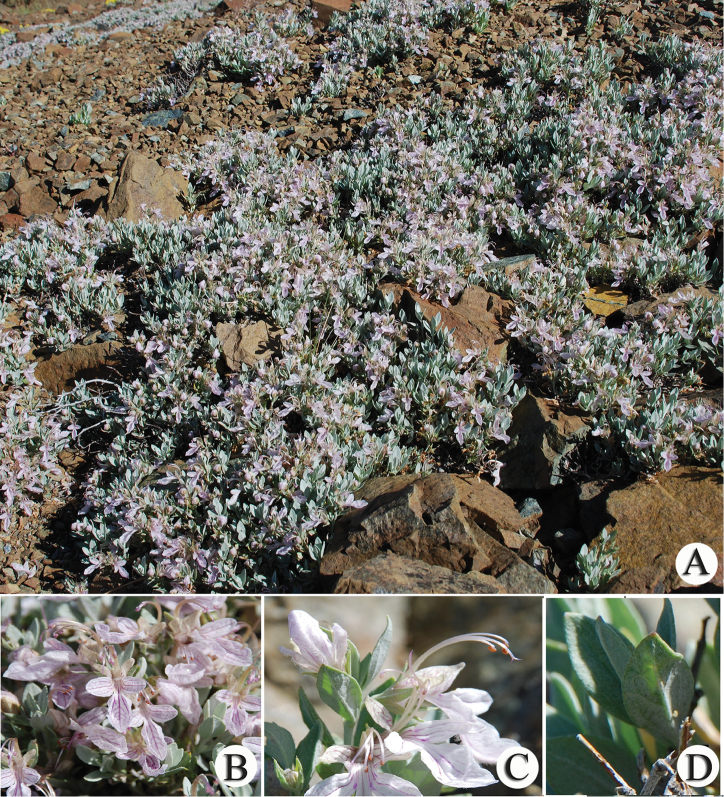
*T. alyssifolium*. **A** habitus **B** inflorescence **C** flower **D** leaf.

*T. pestalozzae* samples were collected from Antalya, *T. brevifolium* and *T. alyssifolium* samples were collected from Muğla provinces in Turkey (Figures 1, 3, 5). Voucher specimens are stored in the Herbarium of the Faculty of Pharmacy, Istanbul University (ISTE). Data about habitats of each investigated species are given in Table 1. Permanent microscopic preparations were made of plant materials fixed in 70% alcohol during the field studies. Cross-sections of the plant leaves and stems were taken manually and stained with Sartur solution (Çelebioğlu and Baytop 1949). Several slides were made and photographed for each species with an Olympus BH-2 and Canon A 640 digital camera.

## Results

**Figure 2.**
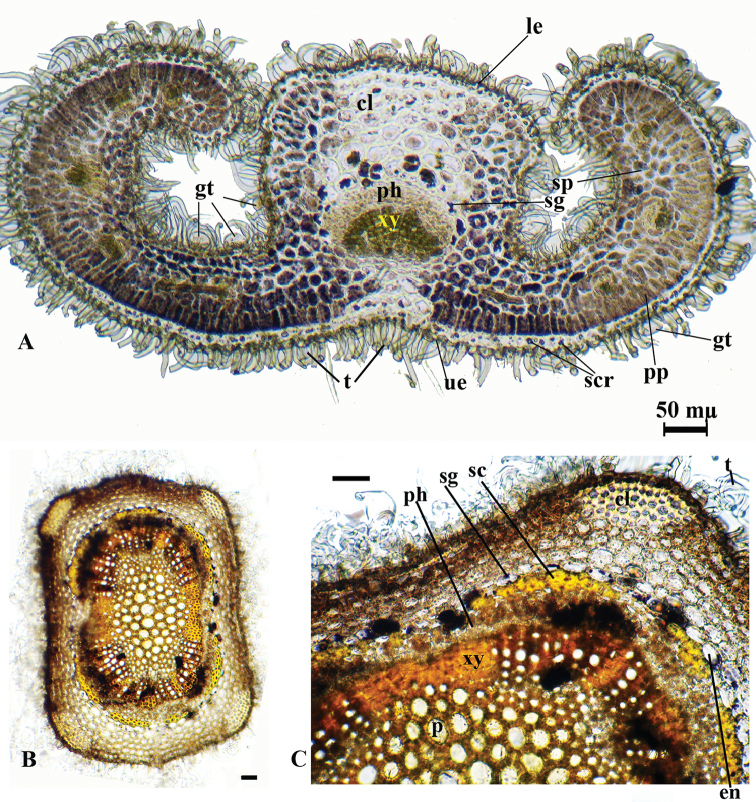
*T. alyssifolium*, cross-section of the leaf (**A**), stem (**B, C**); **cl**: collenchyma; **en**: endodermis; **gt**: glandular trichomes; **le**: lower epidermis; **p**: parenchyma; **ph**: phloem; **pp**: palisade parenchyma; **sc**: sclerenchyma; **scr**: sphaerocrystal, **sg**: starch grains; **sp**: spongy parenchyma; **t**: trichome; **ue**: upper epidermis; **xy**: xylem; Scale bars: 50 mµ.

The anatomy of the collected specimens were assessed by examination of leaf and stem cross sections (Figures 2, 4, 6). This is the first study about the anatomical features of the leaves and stems of *T. alyssifolium*, *T. brevifolium* and *T. pestalozzae*.

### *T. alyssifolium* Stapf

Leaf anatomy

The epidermis at the both surfaces of the leaves is single layered. The epidermis consists of single-layer, ovoid or rectangular cells which are covered by thick cuticula. The upper epidermis cells are larger than the lower ones. Both leaf surfaces are covered by glandular and non-glandular trichomes. Also, the upper epidermis is covered with lower-density trichomes than the lower epidermis. The spherocrystals occur in the upper epidermis cells of the leaf in *T. allysifolium*. Leaves are isolateral. The mesophyll is differentiated into 1 layered palisade and 2–3-layered spongy parenchyma. The palisade parenchyma cells are under the upper and lower epidermis.

Their shapes are cylindrical in transverse section. The palisade parenchyma occupies about 60–65% of the mesophyll. The spongy parenchyma cells, ovoid or circular, are located between the palisade tissues. Both parenchyma tissues contain starch grains. The midrib has 3–4 layered collenchyma and 1–2 layered parenchyma below the lower epidermis. The vascular bundle is located in the central part of the midvein. Vascular bundles are collateral. The xylem layer is just below the collenchyma. 1–2 layered parenchyma and 5–6 layered collenchyma are located under the phloem (Figure 2A).

Stem anatomy

The stem is quadrangular shaped. The epidermis consists of single-layer, ovoid or rectangular cells which are covered by thick cuticula. There are glandular and non-glandular trichomes on the epidermis. Collenchyma with a single layer of cells between the corners but 4–5 layers of collenchyma at the corner of the stem. The cortex, consisting of 3–4 layered ovoidal parenchymatous cells, is located under the collenchyma. The endodermis is conspicuous as a single layer. The vascular tissue is surrounded by 1–2 layers of sclerenchyma fibres. The cambium is indistinguishable. Phloem and xylem members are conspicuous. The pith is present at the middle of the stem and it is completely filled by orbicular parenchymatic cells (Figure 2B, C).

### *Teucrium brevifolium* Schreb.

Leaf anatomy

The epidermis in both surfaces of the leaves is single layered. It is consists of single-layer, ovoid or rectangular cells which are covered by cuticula. Both surfaces are covered by a thick cuticula layer, with dense indumentum built of glandular and non-glandular trichomes. The upper epidermal cells are as large as the lower ones. Spherocrystals are observed in both epidermis cells. Leaves are dorsiventral. Palisade parenchyma has two layers and palisade parenchyma cells shapes are cylindrical in transverse section. The palisade parenchyma occupies about 60% of the mesophyll. Spongy parenchyma consists of four or five layers and their cells are ovoid or circular.

Starch accumulated in both spongy and palisade parenchyma. Midrib has 5–6 layered collenchyma and 1–2 layered parenchyma below the lower epidermis. The collateral vascular bundle is located in the central part of the midvein. The xylem layer is found under the collenchyma. 1–2 layered parenchyma and 2–3 layered collenchyma are located under the phloem (Figure 4A).

Stem anatomy

The stem is rectangular shaped. The epidermis consists of single-layer, ovoid or rectangular cells which are covered by thick cuticula. It is covered by glandular and non-glandular trichomes. Underneath the epidermis, 6–7 layers of collenchyma are located at the corners, 3–4 layered collenchyma is located between the corners. Beneath the collenchyma, 5–6 layered rectangle shaped parenchymatous cells are located. Starch grains are also present in the parenchymatous cells. Endodermis and cambium are inconspicuous. 2–3 sclerenchymatic cell clusters are situated at the corners above the phloem. The pith is present in the middle of the stem and is completely filled by orbicular parenchymatic cells (Figure 4B, C).

### *Teucrium pestalozzae* Boiss.

Leaf anatomy

The epidermis in both surfaces of the leaves is single layered. It is consists of single-layer, ovoid or rectangular cells which are covered by cuticula. The upper epidermis cells are larger than the lower ones. The upper cuticle layer is slightly thicker than the lower ones.

Both surfaces are covered by glandular and non-glandular trichomes. Also, trichomes are abundant on the lower epidermis of leaves and sparse on the upper epidermis of leaves. Leaves are dorsiventral. The spherocrystals occur in the upper epidermis of the mesophyll. Mesophyll is differentiated into 2-layered palisade and 5–6-layered spongy parenchyma. Palisade parenchyma cells are cylindrical shaped in transverse section. The palisade parenchyma occupies about 50–55% of the mesophyll.

The spongy parenchyma cells are ovoid or circular. Both parenchyma tissues densely contain starch grains. Midrib has 5–6 layered collenchyma and 1 layer of parenchyma below the lower epidermis. The collateral vascular bundle is located in the central part of the midvein. The xylem layer is found under the collenchyma. 1–2 layered parenchyma and 4–5 layered collenchyma are located under the phloem (Figure 6A).

Stem anatomy

The stem is rectangular shaped. The epidermis consists of single-layer, rectangular cells which are covered by cuticula. There are glandular and non-glandular trichomes on the epidermis. Underneath the epidermis, there is collenchyma with 1–2 layers between the corners but 7–8 layers of collenchyma at the corner of the stem. The cortex, consisting of 5–6 layers of ovoid shaped parenchymatous cells, is located under the collenchyma. 1–2 layers of sclerenchyma fibres are located above the phloem. The cambium is indistinguishable. Phloem and xylem members are conspicuous. The pith is present in the middle of the stem and is completely composed of orbicular parenchymatic cells (Figure 6B, C).

**Table 1.** Collection data of *Teucrium* taxa studied.

**Taxon**	**Locality, Voucher number (ISTE)**
*T. brevifolium*	Muğla: Marmaris-Knidos, Datça peninsula, 30–100 m elev., 16 May 2012, *T. Özcan, T. Dirmenci, O. Yıldırım*, ISTE 101442.
*T. pestalozzae*	Antalya: Between Antalya and Burdur, Çubuk Beli gateway, 950–1000 m elev., 17 May 2012, *T. Özcan, T. Dirmenci, O. Yıldırım*, ISTE 101448.
*T. alyssifolium*	Muğla: Fethiye-Çameli road, Tuzla Beli gateway, 1440 m elev., 14 April 2011,*T. Özcan, T. Dirmenci, E. Akçiçek*, ISTE 101443.

**Figure 3.**
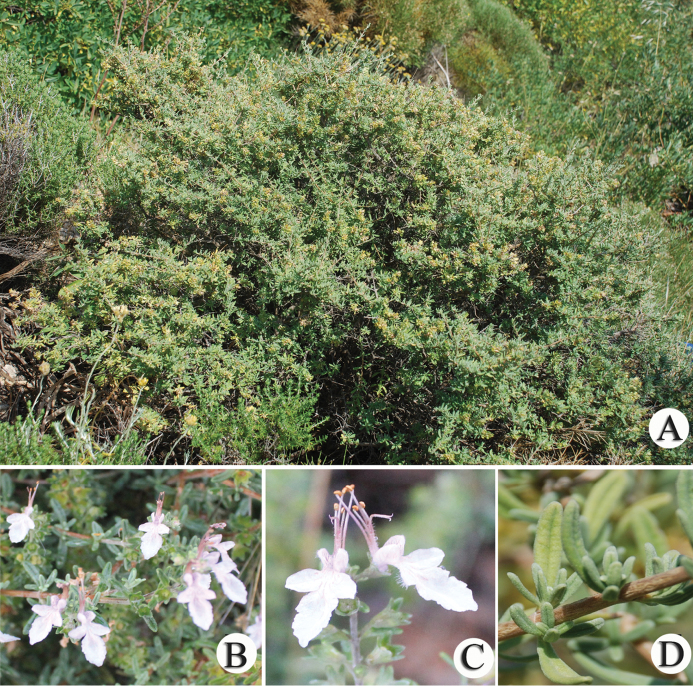
*T. brevifolium*. **A** habitus **B** inflorescence **C** flower **D** leaf.

**Figure 4.**
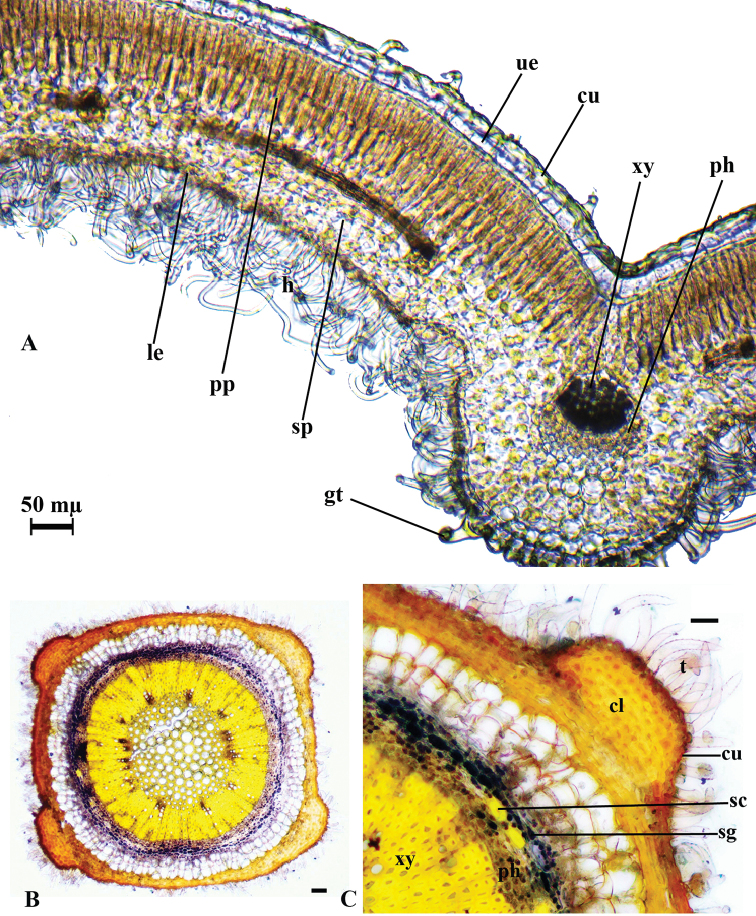
*T. brevifolium*, cross-section of the leaf (**A**), stem (**B, C**); **cl**: collenchyma; **cu**: cuticle; **gt**: glandular trichomes; **le**: lower epidermis; **ph**: phloem; **pp**: palisade parenchyma; **sc**: sclerenchyma; **scr**: sphaerocrystal, **sg**: starch grains; **sp**: spongy parenchyma; **t**: trichome; **ue**: upper epidermis; **xy**: xylem; Scale bars: 50 mµ.

**Figure 5.**
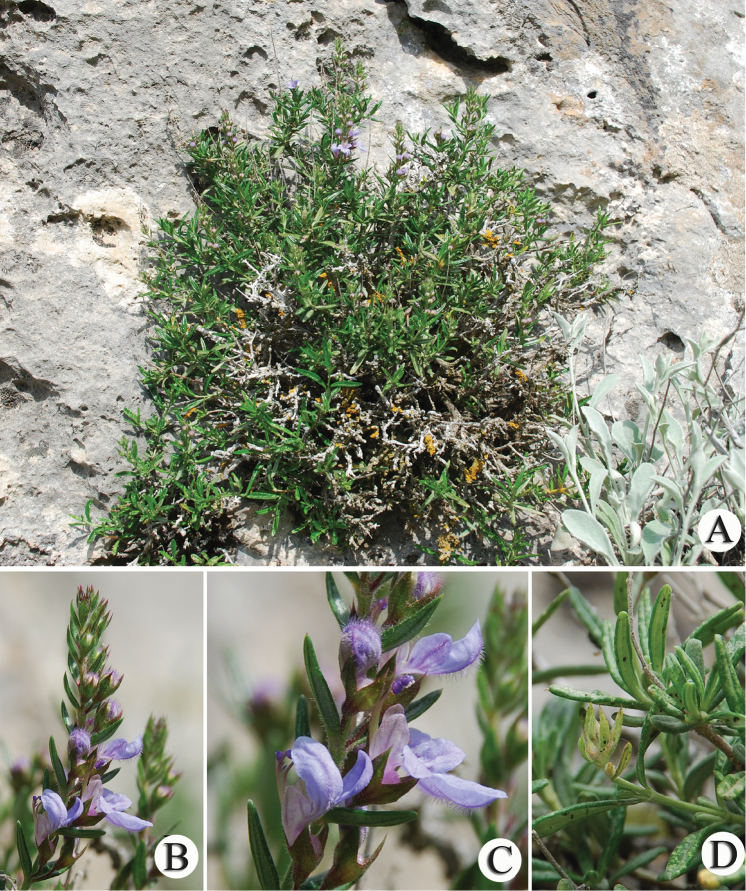
*T. pestalozzae*. **A** habitus **B** inflorescence **C** flower **D** leaf.

**Figure 6.**
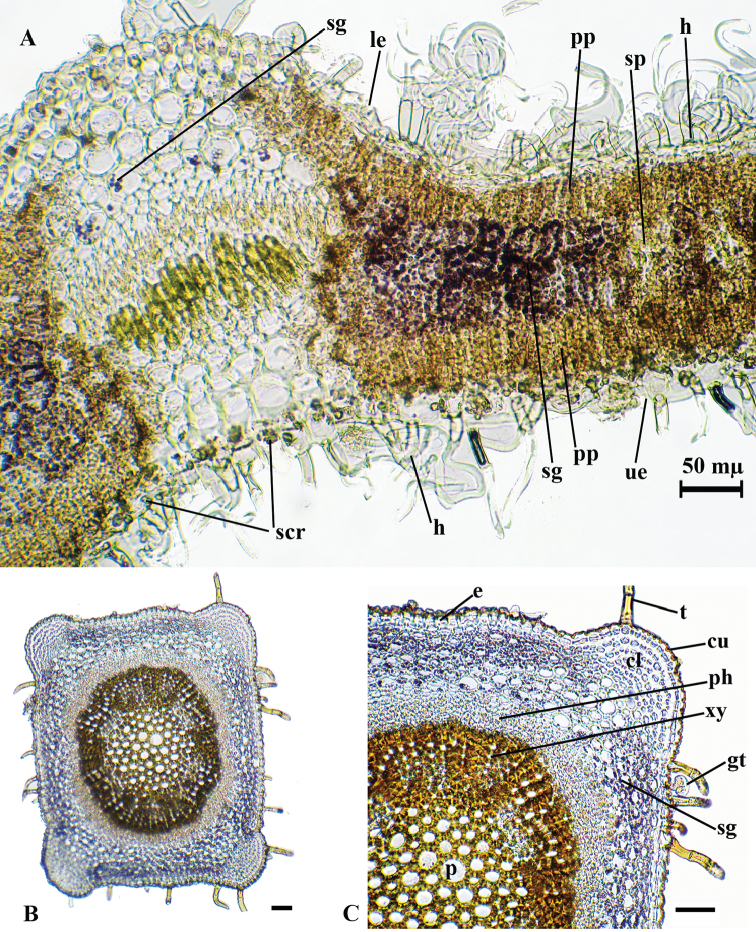
*T. pestalozzae*, cross-section of the leaf (**A**), stem (**B, C**); **cl**: collenchyma; **cu**: cuticle; **e**: epidermis; **gt**: glandular trichomes; **le**: lower epidermis; **p**: parenchyma; **ph**: phloem; **pp**: palisade parenchyma; **sc**: sclerenchyma; **scr**: sphaerocrystal, **sg**: starch grains; **sp**: spongy parenchyma; **t**: trichome; **ue**: upper epidermis; **xy**: xylem; Scale bars: 50 mµ.

**Table 2.** Morphological and anatomical comparison of studied taxa.

		***T. alyssifolium***	***T. brevifolium***	***T. pestalozzae***
**Flowers**		Pedicellate	Pedicellate	Pedicellate
**Pedicels**		2.0–4.0 mm	2.0–12 mm	3.0–5.0 mm
**Bracts**		Orbicular-ovate, oblanceolate	Linear, linear-oblanceolate	Linear-lanceolate
**Bract size**		2.0–20 × 1.5–5.0 mm	3.0–9.0 × 1.0 mm	10–12 mm
**Calyces**		Campanulate	Campanulate	Campanulate
**Calyx size**		6.0–13 × 4.0–5.0 mm	(3.0-) 4.0–5.0 × 2.0–3.5 mm	5.0–6.0 × 3.0–4.0 mm
**Calyx teeth**		4.0–5.0 mm	1.0–2.0 × 1.0 mm	2.5–3.0 mm
**Corolla**		Lilac, light pinkish	White-pinkish	Bluish
**Corolla size**		20–25 mm	6.5–7.0 mm	9.0–9.5 mm
**Filaments**		18– 22 mm	8.0–9.0 mm	7.0–8.0 mm
**Style**		20–22 mm	9.0–10 mm	8.0–9.0 mm
**Leaf**	**Shape**	Orbicular-ovate, oblanceolate	Linear or oblanceolate	Linear, obtuse or oblanceolate
	**Size**	4.0–28 × 2.5–7.0 mm	6.0–17 × 1.0–4.0 mm	11–24 × 2.0–4.0 mm
	**Leaf apex**	Acute or obtuse at apex	Acute or obtuse at apex	Acute or obtuse at apex
	**Cuticular thickness**	Cuticular thickness	Equal	Equal
	**Epidermal cells**	Upper epidermis cells are larger than the lower ones.	Upper epidermal cells are as large as the lower ones	Upper epidermis cells are larger than the lower ones
	**Indumentum density**	Lower surface is denser than upper ones	Same density on both surface	Lower surface is denser than upper ones
	**Mesophyll type**	Isolatheral	Dorsiventral	Dorsiventral
	**Mesophyll**	60–65 % palisade parenchyma	60% palisade parenchyma	60–65 % palisade parenchyma
	**Location of spherocrystals**	Upper epidermis	Both epidermis	Upper epidermis
	**Collenchyma cells layers of midrib**	3–4 layered on the lower surface, 5–6 layered on the upper surface	5–6 layered on the lower surface, 2–3 layered on the upper surface	5–6 layered on the lower surface, 4–5 layered on the upper surface
**Stem**	**Length**	3.5–9.0 cm	30–110 cm	15–18 cm
	**Collenchyma cells layers**	1 layer between the corners, 4–5 layered at the corners	3–4 layers between the corners, 6–7 layers at the corners	1–2 layered between the corners, 7–8 layers at the corners
	**Cortex parenchyma**	3–4 layered	5–6 layered	5–6 layered
	**Endodermis**	Conspicuous	Inconspicuous	Inconspicuous
	**Sclerenchyma**	1–2 layered	2–3 cell clusters	1–2 layered

## Discussion

Sect. Teucrium is one of the eight *Teucrium* sections distributing in Turkey. The members of this section are perennial and shrubs or subshrubs. Leaves are entire to deeply dissected (in *T. orientale* subspecies, *T. parviflorum*, *T. pruinosum*) and revolute at the lower surface.

Flowers borne in racemes or spreading panicles or axillary in upper leaves. Peduncles/pedicels are 1–3-flowered. Calyx not gibbous, obconical-campanulate, teeth ± equal and triangular (Ekim 1982).

Three species showing the characteristic features of the section Teucrium, investigated in the present study, have some significant distinguishing characters. Especially the size of parts of the flowers are very distinctive. *T. alyssifolium* easily differs with its leaf and bract shape, flower, filament and calyx size from *T. brevifolium* and *T. pestalozzae* in their natural habitats (Table 2). *T. alyssifolium* is dwarf, suffruticose and *T. brevifolium* and *T. pestalozzae* are shrublet plants. Stamens of *T. alyssifolium* are longer than its lips, stamens subequal to lip in *T. pestalozzae* and slightly shorter than lips in *T. brevifolium. T. alyssifolium* has the shortest and *T. brevifolium* has the longest stems.

In this study, morphologically related three taxa belonging to the sect. Teucrium have been investigated. Moreover anatomical features of these three species have been reported for the first time. Our results showed the general anatomical characteristics of three *Teucrium* species as well those reported by Metcalfe and Chalk (1950) and Dinç et al. (2008, 2009, 2011a, 2011b), Lakusic et al. (2010), Doğu et al. (2013), Özcan (2013).

The results of the present study revealed that there were differences amongst the leaf anatomy of these three taxa (Table 2). The cuticle layer is on both sides and is of equal thickness to the epidermis for *T. alyssifolium* and *T. brevifolium* leaves. However, the upper cuticle layer of *T. pestalozzae* leaves is slightly thicker than the lower ones. The upper epidermis cells are larger than the lower ones as *T. alyssifolium* and *T. pestalozzae*, but both epidermis cells are the same size as in *T. brevifolium*. *T. alyssifolium* and *T. brevifolium* have a more dense indumentum than *T. pestalozzae*. Also, the indumentum of *T. brevifolium* has the same density on both sides, but the surface of the lower leaves of the other two species is denser than the upper ones. Mesophyll is dorsiventral in *T. brevifolium* and *T. pestalozzae* but isolateral in *T. alyssifolium*. The mesophilic organisation is an important distinguishing character for *T. alyssifolium*. Mesophyll types may be a good distinctive character in different species but sometimes it can be the same in some closer species (Erdoğan et al. 2012).

The palisade parenchyma shows a slight difference in mesophyll amongst the studied taxa. However, these differences can be based on different ecological conditions. Collenchyma layers are different in midrib amongst these studied taxa. The spherocrystals occur in the upper epidermis of the leaf in *T. alyssifolium* and *T. pestalozzae* and both epidermis of the leaf in *T. brevifolium*. According to Metcalfe and Chalk (1950), druse and simple crystals are generally seen in dicotyledon plants. Absence or presence of the crystals and their density are used to distinguish the genera and their species (Salimpour et al. 2009, Güvenç and Kendir 2012). However, spherocrystals and raphides are less common crystal types for dicotyledons. Spherocrystals and raphides have a diagnostic value for dicotyledons (Dinç et al. 2008, 2009, 2013, Ruiters et al. 2016). According to Dinç et al. (2008, 2009) and Ruiters et al. (2016), spherocrystals are an interspecific classification of sect Teucrium. Our results supported their observations.

Some characteristics of the leaf anatomy which indicates of xeromorphy have been reported before in previous studies (Metcalfe and Chalk 1983, Lakusic et al. 2010, Dinç et al. 2008, 2009). According to the results of our study, the three of taxa have leaves with xeromorphic features such as cuticula layer thickness, dense trichomes and a high proportion of the palisade parenchyma in the mesophyll.

In conclusion, this study shows that leaf and stem anatomy have a diagnostic value in the distinction of these three closely related Teucrium species in sect. Teucrium. Anatomical characters contribute to the separation of three species with the morphological characters.

The stem is rectangle shaped in all species. In general, the stems of the family Lamiaceae species are rectangular (Metcalfe and Chalk 1950, Dinç et al. 2008a, 2009, Kahraman et al. 2009, Çalı 2014) or in some genera not (Khalik 2016). However, the stems of the sect. Polium species in Turkey are not conspicuously rectangular. Parenchyma, collenchyma and sclerenchyma layers have some differences amongst the stem of studied taxa. Endodermis is conspicuous only in *T. alyssifolium*. Three studied taxa display general characteristics of Lamiaceae anatomy.

## References

[B1] Aksoy-Sagirli P Ozsoy N Ecevit-Genc G Melikoglu G (2015) In vitro antioxidant activity, cyclooxygenase-2, thioredoxin reductase inhibition and DNA protection properties of *Teucrium sandrasicum* L. Industrial Crops and Products 74: 545–550. https://doi.org/10.1016/j.indcrop.2015.05.025

[B2] Baser KHC Demirçakmak B Duman H (1997) Composition of the essential oils of three *Teucrium* species from Turkey. Journal of Essential Oil Research 9: 545–549. https://doi.org/10.1080/10412905.1997.9700774

[B3] Çalı İÖ (2014) An anatomical study of medicinal species *Ajuga orientalis* L. (Lamiaceae) from Turkey. Journal of Medicinal Plants Research 8(6): 331–338.

[B4] Çelebioğlu S Baytop T (1949) Bitkisel tozların tetkiki için yeni bir reaktif. Farmakolog 19: 301.

[B5] Dehshiri MM Azadbakht M (2012) Anatomy of Iranian species *Teucrium polium* (Lamiaceae) Journal of Biology and Today’s World 1(2): 48–52. https://doi.org/10.15412/J.JBTW.01010204

[B6] Dinç M Dogu S Bilgili B Duran A (2009) Comparative anatomical and micromorphological studies on *Teucrium creticum* and Teucrium orientale var. orientale (Teucrium sect. Teucrium, Lamiaceae). Nordic Journal of Botany 27: 251–256. https://doi.org/10.1111/j.1756-1051.2008.00323.x

[B7] Dinç M Doğu S (2012) Anatomical and micromorphological studies on Teucrium sect. Isotriodon (Lamiaceae) in Turkey with a taxonomic note. Biologia 67(4): 663–672. https://doi.org/10.2478/s11756-012-0049-2

[B8] Dinç M Doğu S Doğru KA Kaya B (2011a) Anatomical and nutlet differentiation between *Teucrium montanum* and *T. polium* from Turkey. Biologia 66(3): 448–453. https://doi.org/10.2478/s11756-011-0035-0

[B9] Dinç M Duran A Pinar M Ozturk M (2008) Anatomy, palynology and nutlet micromorphology of Turkish endemic *Teucrium sandrasicum* (Lamiaceae). Biologia 63(5): 637–641. https://doi.org/10.2478/s11756-008-0137-5

[B10] Dinç M Doğu S Bağcı Y (2011b) Taxonomic reinstatement of *Teucrium andrusi* from *T. paederotoides* based on morphological and anatomical evidences. Nordic Journal of Botany 29: 148–158. https://doi.org/10.1111/j.1756-1051.2011.00894.x

[B11] Dinç M Doğu S (2016) Teucrium pruinosum var. aksarayense var. nov. (Lamiaceae) from Central Anatolia, Turkey. Modern Phytomorphology (9): 13–17.

[B12] Dirmenci T (2012) *Teucrium* L. In: Güner A Aslan S Ekim T Vural M Babaç MT (Eds) Türkiye Bitkileri Listesi (Damarlı Bitkiler). Nezahat Gökyiğit Botanik Bahçesi ve Flora Araştırmaları Derneği Yayını, İstanbul, 595–598.

[B13] Doğu S Dinç M Kaya A Demirci B (2013) Taxonomic status of the subspecies of *Teucrium lamiifolium* in Turkey: Reevaluation based on macro-and micro-morphology, anatomy and chemistry. Nord. J. Bot. 31: 198–207. https://doi.org/10.1111/j.1756-1051.2012.01452.x

[B14] Dönmez AA (2006) *Teucrium chasmophyticum* Rech. f. (Lamiaceae): A new record for the flora of Turkey. Turkish Journal of Botany 30: 317–320.

[B15] Dönmez AA Mutlu B Özçelik AD (2010) *Teucrium melissoides* Boiss. & Hausskn. ex Boiss. (Lamiaceae): A new record for Flora of Turkey. Hacettepe Journal of Biology and Chemistry 38: 291–294.

[B16] Duman H (2000) *Teucrium* L. In: Güner A Özhatay N Ekim T Başer KHC (Eds) Flora of Turkey and East Aegean Islands (Suppl. II). Edinburgh University Press, 197–198.

[B17] Ekim T (1982) *Teucrium* L. In: Davis PH (Ed.) Flora of Turkey and the East Aegean Islands, Vol. 7. Edinburgh University Press, Edinburgh, 53–75.

[B18] Ecevit-Genc G Ozcan T Dirmenci T (2017) Nutlet and leaf micromorphology in some Turkish species of *Teucrium* L. (Lamiaceae). Phytotaxa 312(1): 71–82. https://doi.org/10.11646/phytotaxa.312.1.5

[B19] Ecevit-Genc G Özcan T Dirmenci T (2015) Micromorphological characters on nutlet and leaf indumentum of Teucrium sect. Teucrium (Lamiaceae) in Turkey. Turkish Journal of Botany 39: 439–448. https://doi.org/10.3906/bot-1406-18

[B20] Erdoğan E Akçiçek E Selvi S Tümen G (2012) Comparative anatomical studies on the two Stachys species (sect. Eriostomum, subsect. Germanicae) growing in Turkey. African Journal of Pharmacy and Pharmacology 6(19): 1417–1427. https://doi.org/10.5897/AJPP12.267

[B21] Govaerts R (1999) World Checklist Seed Plants 3. Continental Publishing, Deurne, 1532.

[B22] Güvenç A Kendir G (2012) The leaf anatomy of some *Erica* taxa native to Turkey. Turkish Journal of Botany 36(3): 253–262.

[B23] Kahraman A Celep F Doğan M (2009) Comparative morphology, anatomy and palynology of two *Salvia* L. species (Lamiaceae) and their taxonomic implications. Bangladesh Journal of Plant Taxonomy 16(1): 73–82. https://doi.org/10.3329/bjpt.v16i1.2749

[B24] Khalik KA (2016) A new species of *Plectranthus* (Lamiaceae) from Saudi Arabia. Turkish Journal of Botany 40: 506–513. https://doi.org/10.3906/bot-1601-8

[B25] Lakusic B Lakusic D Jancic R Stevanovic B (2006) Morpho-anatomical differentiation of the Balkan populations of the species *Teucrium flavum* L. (Lamiaceae). Flora 201: 108–119. https://doi.org/10.1016/j.flora.2005.05.001

[B26] Lakusic B Stevanovic B Jancic R Lakusic D (2010) Habitat-related adaptations in morphology and anatomy of *Teucrium* (Lamiaceae) species from Balkan peninsula (Serbia and Montenegro). Flora 205: 633–646. https://doi.org/10.1016/j.flora.2010.04.018

[B27] Marzouk RI Salama M El-Darier M Askar ABM (2017) Pollen morphology of *Teucrium* L. (Lamiaceae, Ajugoideae) in Libya. Bangladesh Journal of Plant Taxonomy 24(2): 219–226. https://doi.org/10.3329/bjpt.v24i2.35118

[B28] Menichini F Conforti F Rigano D Formisano C Piozzi F Senatore F (2009) Phytochemical composition, anti-inflammatory and antitumour activities of four *Teucrium* essential oils from Greece. Food Chemistry 115(2): 15 679–686.

[B29] Metcalfe CR Chalk L (1950) Anatomy of the Dicotyledons I. Oxford University Press, London, 1041–1053.

[B30] Navarro T El Oualidi J Trigo MM (2004) Pollen morphology of *Teucrium* (Labiatae) and its taxonomic value. Belgian Journal of Botany 137(1): 70–84.

[B31] Oybak E İnceoğlu Ö (1988) Pollen morphology of some *Teucrium* L. (Labiatae) species. Communications Faculty of Sciences University of Ankara Series C: Biology 6: 133–146. https://doi.org/10.1501/Commuc_0000000133

[B32] Özcan M Eminagaoglu O (2014) Stem and leaf anatomy of three taxa in Lamiaceae. Pakistan Journal of Botany 43(3): 345–352.

[B33] Özcan T (2013) Presence of *Teucrium microphyllum* in Turkey: Morpho-anatomical, karyological and ecological studies. Biodicon 6: 79–87.

[B34] Özcan T Dirmenci T Coskun F Akcicek E Güner Ö (2015a) A new species of Teucrium sect. Scordium (Lamiaceae) from SE of Turkey. Turkish Journal of Botany 39: 310–317. https://doi.org/10.3906/bot-1402-93

[B35] Özcan T Dirmenci T Martin E Altinordu F (2015b) Cytotaxonomical study in five taxa of the genus *Teucrium* L. (Lamiaceae). Caryologia 68(1): 1–8. https://doi.org/10.1080/00087114.2014.996037

[B36] Parolly G Eren Ö (2007) Contributions to the flora of Turkey. 2. Willdenowia 37: 245–246. https://doi.org/10.3372/wi.37.37114

[B37] Ruiters AK Tilney PM Van Vuuren SF Viljoen AM Kamatou GPP VanWyk BE (2016) The anatomy, ethnobotany, antimicrobial activity and essential oil composition of southern African species of *Teucrium* (Lamiaceae). South African Journal of Botany 102: 175–185. https://doi.org/10.1016/j.sajb.2015.06.008

[B38] Salimpour F Mazooji A Onsori S (2009) Stem and leaf anatomy of ten *Geranium* L. species in Iran. African Journal of Plant Science 3(11): 238–244.

[B39] Semiz G Çelik G Gönen E Semiz A (2016) Essential oil composition, antioxidant activity and phenolic content of endemic *Teucrium alyssifolium* Staph. (Lamiaceae). Natural Product Research 30(19): 2225–2229. https://doi.org/10.1080/14786419.2016.1149703 26918276 10.1080/14786419.2016.1149703

[B40] Sezik E Yeşilada E Honda G Takaishi Y Takeda Y Tanaka T (2001) Traditional medicine in Turkey X. Folk medicine in Central Anatolia. Journal of Ethnopharmacology 75: 95–115. https://doi.org/10.1016/S0378-8741(00)00399-8 11297840 10.1016/s0378-8741(00)00399-8

[B41] Vural M Duman H Dirmenci T Özcan T (2015) A new species of Teucrium Sect. Stachyobotrys (Lamiaceae) from South of Turkey. Turkish Journal of Botany 39: 318–324. https://doi.org/10.3906/bot-1403-50

